# Analgesic Efficacy of Remifentanil Versus Dexmedetomidine in Patients Undergoing Bariatric and Metabolic Surgeries: A Systematic Review and Meta-Analysis of Randomized Controlled Trials

**DOI:** 10.7759/cureus.86691

**Published:** 2025-06-24

**Authors:** Abhijit Nair, Tuhin Mistry, Hosam Elghadban, Chetan Shende, Nitinkumar Borkar, Ahmed Elzayyat

**Affiliations:** 1 Anesthesiology, Ibra Hospital, Ibra, OMN; 2 Anesthesiology, Ganga Medical Centre and Hospitals Pvt. Ltd., Coimbatore, IND; 3 General Surgery, Ibra Hospital, Ibra, OMN; 4 Pediatric Medicine, Colours Hospital, Nagpur, IND; 5 Pediatric Surgery, All India Institute of Medical Sciences, Raipur, Raipur, IND; 6 Intensive Care Unit, Rashid Hospital, Dubai, ARE

**Keywords:** `anesthesia, bariatric & metabolic surgery, peri-operative analgesia, remifentanil, dexmedetomidine

## Abstract

Obese patients undergoing bariatric and metabolic surgeries require tailored perioperative pain management. This review aimed to compare the analgesic efficacy and safety of two adjuncts used in general anesthesia (GA), remifentanil and dexmedetomidine, in this patient population. Using relevant keywords, we searched PubMed, Scopus, the Cochrane Library, and ClinicalTrials.gov, identifying five randomized controlled trials for a qualitative systematic review and quantitative meta-analysis. The RoB 2 tool was used to assess the risk of bias, and the meta-analysis was conducted using RevMan version 5.4. The Grading of Recommendations, Assessment, Development, and Evaluation (GRADE) approach was employed to evaluate the overall quality of evidence. Trial Sequential Analysis (TSA) was used to confirm significant findings. The overall risk of bias was low, and the GRADE quality ranged from moderate to low. Twenty-four-hour opioid consumption and pain scores in the recovery room were comparable between the two groups (mean difference (MD): 0.23; 95% CI: -1.42 to 1.89, P = 0.78; and MD: 0.04; 95% CI: -0.48 to 0.57, P = 0.87, respectively). Postoperative nausea and vomiting (PONV) was significantly lower in the dexmedetomidine group (OR: 2.55; 95% CI: 1.60 to 4.07, P < 0.0001), a finding confirmed by TSA. However, the cumulative sample size represented only 82.5% of the required information size. Overall heterogeneity was low to moderate. Based on the findings of this review, analgesic efficacy, measured by 24-hour opioid consumption and recovery room pain scores, appears comparable between remifentanil and dexmedetomidine. However, the incidence of PONV was significantly lower in the dexmedetomidine group. Further studies are warranted to identify the most suitable adjunct to GA in this high-risk patient population.

## Introduction and background

Obesity affects pharmacokinetics, pharmacodynamics, and respiratory physiology, presenting specific challenges in anesthesia management. Altered drug distribution and opioid elimination in obese patients can lead to respiratory depression and prolonged sedation if not used judiciously. This necessitates monitoring in high-dependency areas, potentially delaying recovery and early mobilization [1]. The volume of distribution for lipophilic opioids, such as fentanyl, increases due to excess adipose tissue, leading to drug accumulation and prolonged effects [2]. In contrast, hydrophilic opioids like remifentanil exhibit a more predictable pharmacokinetic profile in patients undergoing bariatric and metabolic surgeries, as their rapid metabolism remains unaffected by body fat [3].

Dexmedetomidine, a selective alpha-2 adrenergic agonist, possesses analgesic, sedative, and opioid-sparing properties. Its sympatholytic effects lower blood pressure and heart rate without causing significant respiratory depression, making it particularly beneficial for obese patients [4]. By reducing opioid requirements and maintaining airway reflexes and spontaneous breathing, dexmedetomidine facilitates opioid-sparing anesthesia and minimizes the risk of respiratory depression [5].

Remifentanil, a unique opioid metabolized by nonspecific esterases, offers a rapid and predictable onset and offset of action due to its quick clearance [6]. Although its ultra-short half-life accelerates postoperative recovery, alternative analgesia is required postoperatively [7]. In patients with obstructive sleep apnea (OSA) or opioid sensitivity, dexmedetomidine enhances postoperative pain management, reduces opioid requirements, and improves the quality of recovery. Dexmedetomidine is primarily metabolized by hepatic enzymes, and its clearance may be affected by changes in regional blood flow and hepatic function in obesity. Since OSA is highly prevalent in obese patients, opioid-induced respiratory depression remains a major concern [8]. Despite its rapid metabolism, remifentanil still carries a limited but present risk of respiratory depression. In contrast, dexmedetomidine reduces opioid requirements, preserves airway reflexes, and supports spontaneous breathing, offering an added advantage in minimizing respiratory complications.

Several studies have compared opioids with opioid-sparing strategies, such as dexmedetomidine, in patients undergoing bariatric surgeries [9,10]. This review aimed to compare the analgesic efficacy of remifentanil and dexmedetomidine, along with other outcomes including pain scores, adverse events, and anesthesia and surgical duration, when used as adjuncts to general anesthesia (GA) in patients undergoing bariatric and metabolic surgeries.

## Review

Materials and methods

This systematic review and meta-analysis were conducted in accordance with the Preferred Reporting Items for Systematic Reviews and Meta-Analyses (PRISMA) guidelines. The review was prospectively registered with PROSPERO (CRD420251002300) at https://www.crd.york.ac.uk/PROSPERO.

Search Strategy

We searched major databases (PubMed, Scopus, Embase, Cochrane Library) and an additional clinical trial registry (ClinicalTrials.gov). We sought randomized controlled trials (RCTs) comparing remifentanil with dexmedetomidine as adjuncts to anesthesia using relevant keywords, covering the period from January 2000 to February 2025. The details of the search strategy and truncations are provided in Table 1. The primary outcome was the comparison of 24-hour opioid consumption. The secondary outcomes included the comparison of adverse events like postoperative nausea/vomiting (PONV), hemodynamic and respiratory adverse events, pain scores, and surgical and anesthesia time.

**Table 1:** Databases searched and truncations.

Database	Truncations
PubMed	Search: ((dexmedetomidine) AND (remifentanil)) AND (bariatric surgery) Sort by: Most Recent. ("dexmedetomidine"[MeSH Terms] OR "dexmedetomidine"[All Fields] OR "dexmedetomidine's"[All Fields]) AND ("remifentanil"[MeSH Terms] OR "remifentanil"[All Fields] OR "remifentanil's"[All Fields]) AND ("bariatric surgery"[MeSH Terms] OR ("bariatric"[All Fields] AND "surgery"[All Fields]) OR "bariatric surgery"[All Fields]) Translations: dexmedetomidine: "dexmedetomidine"[MeSH Terms] OR "dexmedetomidine"[All Fields] OR "dexmedetomidine's"[All Fields] remifentanil: "remifentanil"[MeSH Terms] OR "remifentanil"[All Fields] OR "remifentanil's"[All Fields] bariatric surgery: "bariatric surgery"[MeSH Terms] OR ("bariatric"[All Fields] AND "surgery"[All Fields]) OR "bariatric surgery"[All Fields]
Scopus	dexmedetomidine AND remifentanil AND bariatric AND surgery AND PUBYEAR > 2003 AND PUBYEAR < 2026 AND (EXCLUDE (SUBJAREA, "VETE") OR EXCLUDE (SUBJAREA, "CENG") OR EXCLUDE (SUBJAREA, "PSYC") OR EXCLUDE (SUBJAREA, "MATE") OR EXCLUDE (SUBJAREA, "ENVI") OR EXCLUDE (SUBJAREA, "AGRI")) AND (EXCLUDE (LANGUAGE, "Spanish") OR EXCLUDE (LANGUAGE, "German") OR EXCLUDE (LANGUAGE, "Chinese") OR EXCLUDE (LANGUAGE, "Turkish") OR EXCLUDE (LANGUAGE, "French") OR EXCLUDE (LANGUAGE, "Japanese") OR EXCLUDE (LANGUAGE, "Russian") OR EXCLUDE (LANGUAGE, "Ukrainian") OR EXCLUDE (LANGUAGE, "Persian") OR EXCLUDE (LANGUAGE, "Greek") OR EXCLUDE (LANGUAGE, "Czech") OR EXCLUDE (LANGUAGE, "Bulgarian") OR EXCLUDE (LANGUAGE, "Portuguese")) AND (EXCLUDE (DOCTYPE, "ch") OR EXCLUDE (DOCTYPE, "le") OR EXCLUDE (DOCTYPE, "bk") OR EXCLUDE (DOCTYPE, "sh"))

Eligibility Criteria

To identify studies that are relevant, we employed a structured Population, Intervention, Control, Outcome, and Study (PICOS) design. The quantitative analysis was confined to RCTs. Studies performed in languages other than English, research involving animals, studies for which full texts were not available, systematic reviews, literature reviews, scoping reviews, case reports, series, editorials, and conference abstracts were excluded.

Population: All adult patients undergoing elective bariatric and metabolic surgeries under GA were included.

Intervention: Use of intraoperative infusion of remifentanil.

Control: Use of intraoperative infusion of dexmedetomidine.

Outcome: Analgesic efficacy, adverse events, surgery, and anesthesia time.

Study: RCTs

Data Extraction

Two authors (AN and TM) independently screened the identified studies based on the inclusion and exclusion criteria. A third author (HE) was asked to resolve any disagreements regarding study inclusion. Once the articles were finalized, their details were summarized in a table under the following headings: author and year of publication, country, type of study, number of participants per intervention arm, description of intervention and control, and primary and secondary outcomes.

Methodological Assessment

Using the Risk of Bias (ROB2) tool and the Cochrane Intervention System Evaluation Manual (Cochrane Handbook for Systematic Reviews of Interventions), two authors (AN and TM) independently reviewed the methodological quality of the articles that fulfilled the inclusion criteria [11]. If these two researchers disagreed on the methodological evaluation, a third researcher (HE) was involved to resolve the discrepancy and make a decision. Bias resulting from the randomization process, bias resulting from deviations from the intended intervention, bias resulting from missing outcome data, bias in outcome measurement, and bias in the selection of the reported result were all included in the methodological assessment (ROB2).

Strength of Quality Across All Trials

The Grading of Recommendations, Assessment, Development, and Evaluation (GRADE) guidelines were adopted to assess the methodological quality of the evidence across pooled outcomes [12]. The study design, ROB, consistency, directness, precision, and additional factors including confounding, large effect size, and publication bias were taken into account when establishing the quality of evidence for pooled outcomes. The certainty of the evidence was categorized as follows: (1) high quality, additional research is improbable to alter the confidence in the estimate of effect; (2) moderate quality, additional research is likely to affect the estimate’s confidence significantly and may alter it; (3) low quality, additional research is highly likely to alter the estimate; or (4) very low quality, the estimate is uncertain.

Quantitative Meta-Analysis

We used Review Manager software (RevMan 5.4) by the Cochrane Collaboration for conducting the meta-analysis [13]. The binary variables were reported using OR and 95% CI, and the continuous variables with mean difference (MD) and 95% CI. The effect estimates were derived from forest plot analysis comparing similar groups. A p-value less than 0.05 was regarded as statistically significant. Inconsistencies were evaluated using the χ² and I² statistics to assess the clinical heterogeneity in the included studies, and p < 0.10 and I² > 50% indicated that χ² had statistical differences [14]. A fixed-effects model was utilized if the study results showed heterogeneity, i.e., I² < 50% and p > 0.10. The random-effects model was utilized for the meta-analysis in all other cases [15]. For comparison purposes between the trials, different opioids were converted to IV morphine equivalent (https://www.mdcalc.com/calc/3947/opiate-conversion-calculator).

Sensitivity Analysis

Each study was sequentially removed from the analysis to rule out any particular study exerting greater influence on the interpretation of the results [16]. A funnel plot was constructed to determine if there was publication bias [17].

Trial Sequential Analysis (TSA)

We planned a TSA for those outcomes that were statistically significant in quantitative meta-analysis using the TSA Module version 0.9.5.10 (Copenhagen Trial Unit, Denmark) to calculate the required information size (RIS) and see if our findings were conclusive. The DerSimonian-Laird (DL) method was used to create the cumulative Z-curve. The TSA was carried out to keep the overall risk of a Type I error at 5% [18].

Results

**Figure 1:**
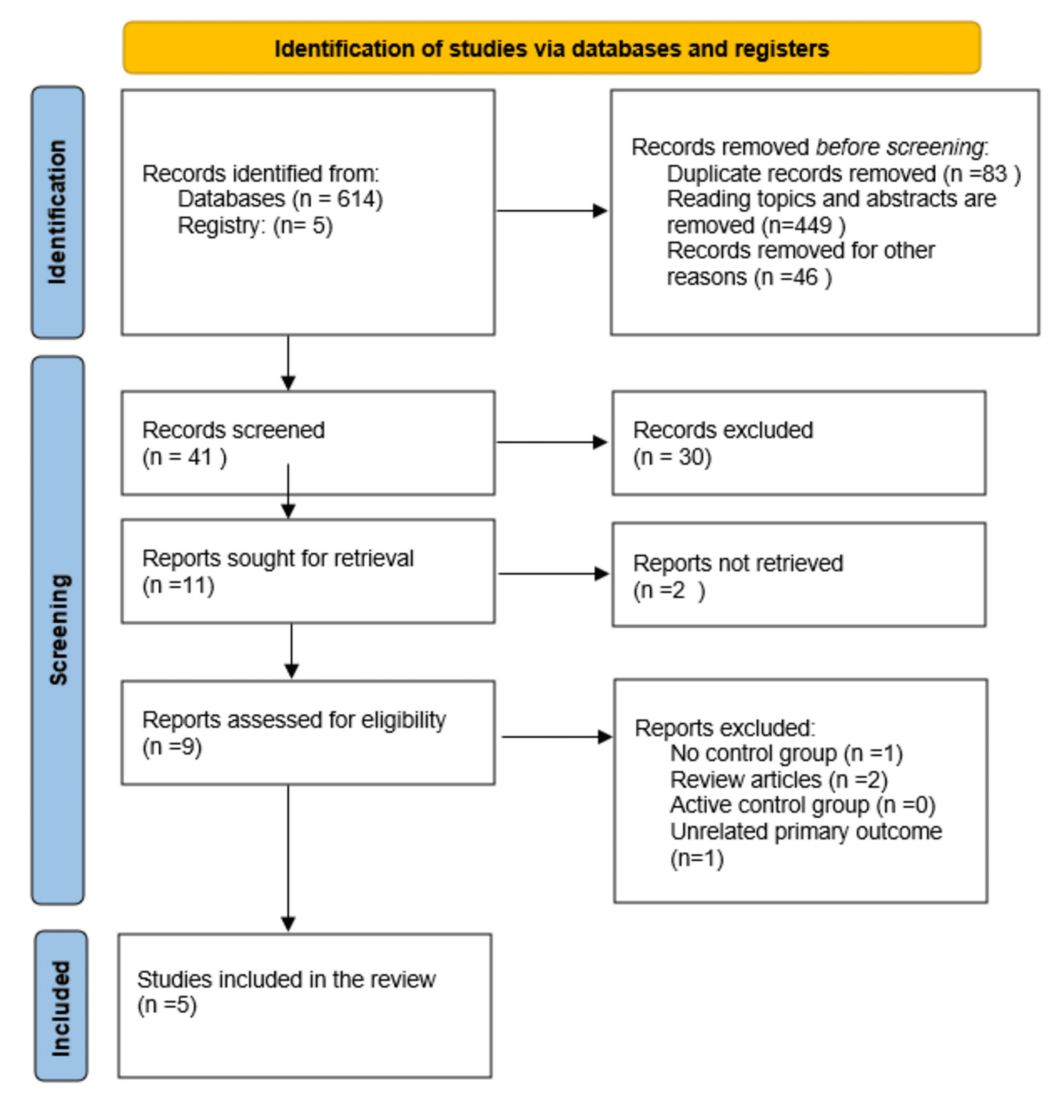
PRISMA flow diagram. PRISMA: Preferred Reporting Items for Systematic Reviews and Meta-Analyses.

We identified 614 articles based on the inclusion criteria mentioned above. After removing duplicates and excluding articles that were not relevant, 41 titles were screened, out of which 30 were excluded. From the remaining 11 articles, 2 articles were not retrieved as they were not relevant. Of the remaining 9 articles, 4 were excluded (one with no control group, two review articles, and one with unrelated primary outcomes). Finally, 5 RCTs were selected for a qualitative systematic review and quantitative meta-analysis [19-23]. The PRISMA flow diagram is presented in Figure 1. Study characteristics and outcome details are summarized in Tables 2-3.

**Table 2:** Summary of all included studies. PONV: Postoperative nausea/vomiting; PACU: Post-anesthesia care unit; NRS: Numerical rating scale; LOS: Length of stay.

S. No.	Author(s) and Year	Country	Study Period	Number of Patients	Intervention	Control	Reported Outcomes
1	Mieszczański P et al. [19] (2023)	Poland	February 2020-October 2022	Interventional: 29 Control: 30	Remifentanil	Opioid-free anesthesia (dexmedetomidine and lidocaine infusion)	Primary outcomes were total oxycodone consumption and pain scores determined using the NRS scale at 1, 6, 12, and 24 hours after surgery. Other outcomes: Ramsay Sedation Scale, PONV, desaturation, pruritus (at 1, 6, 12, and 24 h), and hemodynamic variables.
2	Clanet M et al. [20] (2024)	Belgium	Not reported	Interventional: 86 Control: 86	Remifentanil	Dexmedetomidine	Primary outcome: 24-hour morphine consumption. Secondary outcomes: quality of recovery, incidence of hypoxemia, bradycardia, and PONV.
3	Hamed JM et al. [21] (2019)	Saudi Arabia	June 2017 - March 2018	Interventional: 66 Control: 66	Remifentanil	Dexmedetomidine	Primary outcome: ability to reduce postoperative pethidine use to one dose within 24 hours. Secondary outcomes: 24-hour narcotic use, PONV, and time to rescue analgesia.
4	Narejo AS et al. [22] (2021)	Saudi Arabia	December 2019 - March 2020	Interventional: 20 Control: 20	Remifentanil	Dexmedetomidine	Primary outcome: evaluation of postoperative pain. Secondary outcomes: cumulative morphine consumption, duration of surgery, time to awakening and tracheal extubation, Sedation Agitation Scale (SAS), shivering, PONV, and LOS.
5	Barakat H et al. [23] (2025)	Lebanon	March 2018 - December 2020	Interventional: 43 Control: 40	Remifentanil	Dexmedetomidine + lidocaine	Primary outcome: postoperative morphine consumption in PACU. Secondary outcomes: duration of stay, 24- and 48-hour morphine consumption, pain score at discharge, PONV, and use of rescue analgesia.

**Table 3:** Table summarizing outcomes analyzed in the quantitative meta-analysis. R: Remifentanil; D: Dexmedetomidine; PONV: Postoperative nausea/vomiting.

S.no.	Study	R/D	Number of patients	24-hour opioid consumption (mg)	Pain scores	PONV	Surgery duration (minutes)	Anesthesia duration (minutes)
1	Mieszczański P et al. [19] (2023)	R	29	18.26 +/- 11.7	4.06 +/-2.64	1/29	89.41 +/- 25.5	107.38 +/- 22.8
		D	30	25.05 +/-11.26	3.57 +/- 3.05	0/30	86.27 +/- 27.4	108.9 +/- 26.5
2	Clanet M et al. [20] (2024)	R	86	15 +/- 18.9	4 +/- 1.35	51/86	---	---
		D	86	16 +/- 17.55	4 +/- 2.7	32/86	---	---
3	Hamed JM et al. [21](2019)	R	66	12.2 +/- 0	0.27 +/- 0.45	10/66	---	---
		D	66	10.7 +/- 0	0 +/- 0	3/66	---	---
4	Narejo AS et al. [22] (2021)	R	20	11. 05 +/- 4.12	4.15 +/-1.9	6/20	63.25 +/- 16.18	---
		D	20	10.45 +/- 4.29	4.26 +/- 1.97	1/20	74.6 +/- 23.19	---
5	Barakat H et al. [23] (2025)	R	43	7.28 +/- 6.02	---	12/43	128.35 +/ 38.97	163.49 +/ 44.43
		D	40	5.79 +/- 5.82	---	8/20	124.58 +/ 31.79	165.35 +/- 37.76

Qualitative Systematic Review

**Figure 2:**
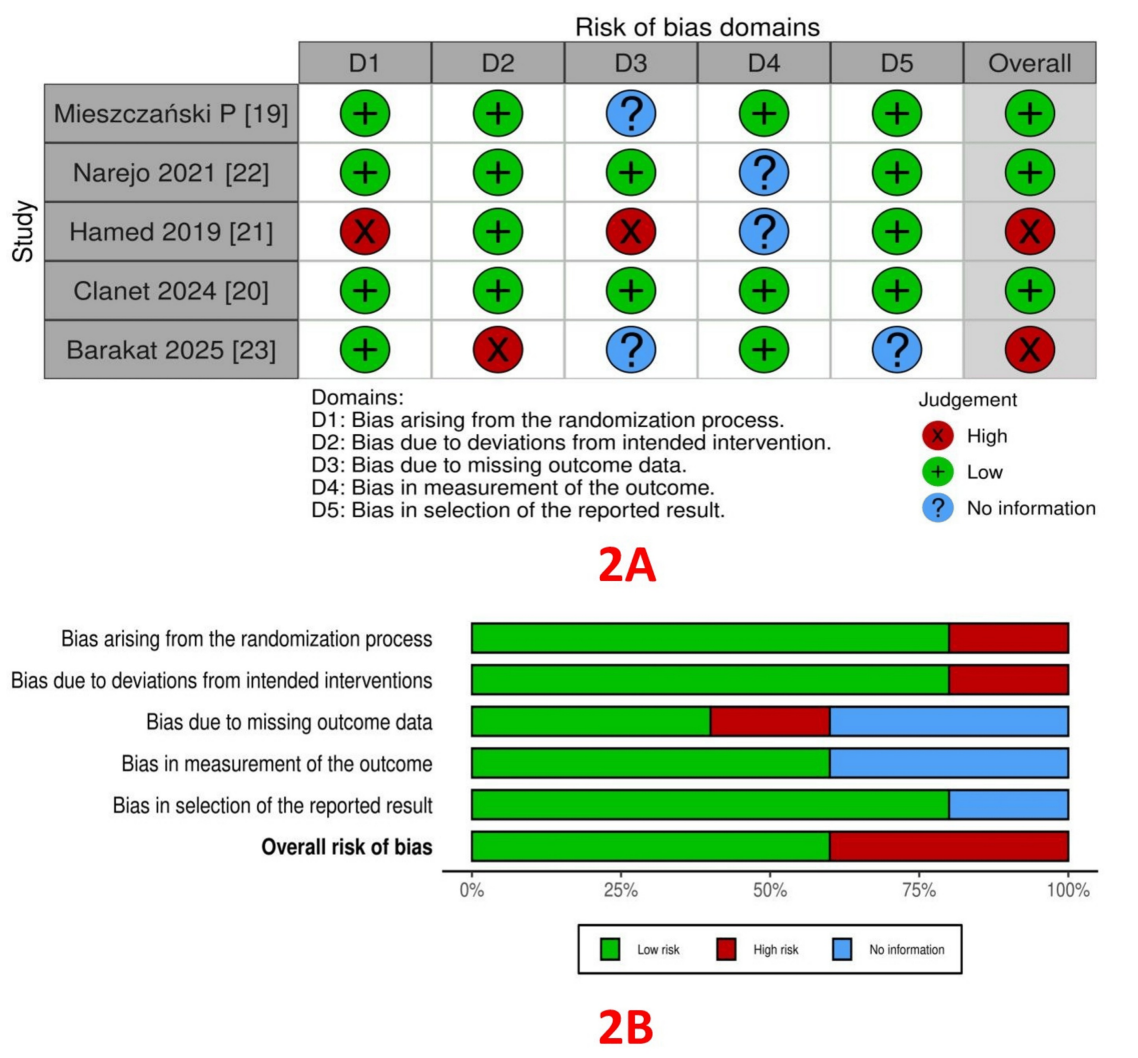
A: Traffic light plot; B: Summary plot.

RoB assessment: The RoB within the trials, assessed using the ROB2 tool, is depicted in Figure 2A (traffic light plot) and Figure 2B (summary plot). Bias from the randomization process was low in 4 studies [19, 20, 22, 23] and high in one study [21]. Bias due to deviations from intended interventions (allocation concealment) was low in four studies [19-22] and high in one study [23]. Bias arising from missing outcome data was low in two studies [20, 22], high in one study [21], and not reported in two studies [19, 23]. Bias in outcome measurement was low in three studies [19, 20, 23], and not reported in two studies [21, 22]. Bias arising from the selection of reported results was low in four studies [19-22] and high in one study [23].

Quality of evidence: GRADE assessment was performed for three outcomes - 24-hour opioid consumption, pain scores, and PONV (Table 4). The level of evidence was low for 24-hour opioid consumption and PONV, and moderate for pain scores.

**Table 4:** GRADE level of evidence. MD: Mean difference; PONV: Postoperative nausea and vomiting. Explanations: a. Risk of bias due to randomization was high in one study. b. Publication bias was noted. c. Bias due to missing outcome data and outcome measurement was observed.

Certainty assessment							No. of patients		Effect		Certainty
No. of studies	Study design	Risk of bias	Inconsistency	Indirectness	Imprecision	Other considerations	Remifentanil	Dexmedetomidine	Relative (95% CI)	Absolute (95% CI)	
24-hour opioid consumption											
5	Randomised trials	Serious^a^	Not serious	Not serious	Not serious	Publication bias strongly suspected^b^	244	242	-	MD 0.23 higher (1.42 lower to 1.89 higher)	⨁⨁◯◯ Low^a,b^
Pain scores											
4	Randomised trials	Serious^c^	Not serious	Not serious	Not serious	None	201	202	-	MD 0.04 higher (0.48 lower to 0.57 higher)	⨁⨁⨁◯ Moderate^c^
PONV											
5	Randomised trials	Serious^a,c^	Not serious	Not serious	Not serious	Publication bias strongly suspected^b^	80/244 (32.8%)	44/242 (18.2%)	OR 2.55 (1.60 to 4.07)	180 more per 1,000 (from 80 more to 293 more)	⨁⨁◯◯ Low^a,b,c^

Quantitative Meta-Analysis

**Figure 3:**
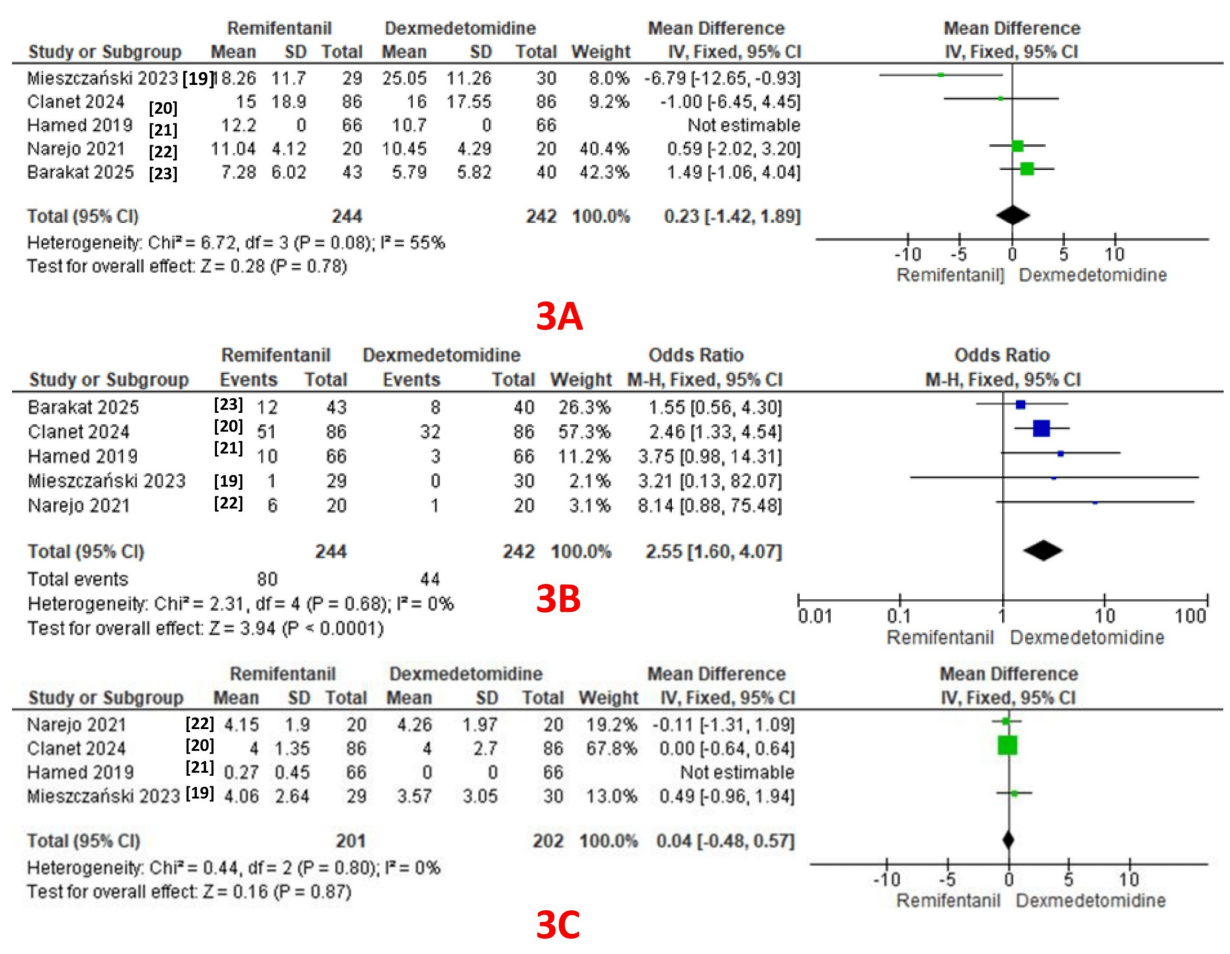
A: Forest plot comparing 24-hour opioid consumption; B: Forest plot comparing PONV; C: Forest plot comparing pain scores in the recovery room. PONV: Postoperative nausea/vomiting.

Twenty-four-hour opioid consumption was reported as an outcome in all five studies (244 patients in the remifentanil group and 242 patients in the dexmedetomidine group) [19-23]. A pooled analysis revealed comparable 24-hour opioid consumption in both groups (MD: 0.23; 95% CI: -1.42 to 1.89, P = 0.78). A fixed-effect model revealed moderate heterogeneity (I² = 55%) (GRADE = low) (Figure 3A).

PONV: PONV was reported as an outcome in all five studies (244 patients in the remifentanil group and 242 patients in the dexmedetomidine group), with 80 events in the remifentanil group and 44 in the dexmedetomidine group [19-23]. A pooled analysis revealed a significantly lower incidence of PONV in the dexmedetomidine group (OR: 2.55; 95% CI: 1.60 to 4.07, P < 0.0001). A fixed-effect model revealed no heterogeneity (I² = 0%) (GRADE = low) (Figure 3B).

Pain scores in the recovery room: Four studies reported pain scores as an outcome (201 patients in the remifentanil group and 202 patients in the dexmedetomidine group) [19-22]. A pooled analysis revealed comparable pain scores in both groups (MD: 0.04; 95% CI: -0.48 to 0.57, P = 0.87). A fixed-effect model revealed no heterogeneity (I² = 0%) (GRADE = moderate) (Figure 3C).

Surgery Duration

**Figure 4:**
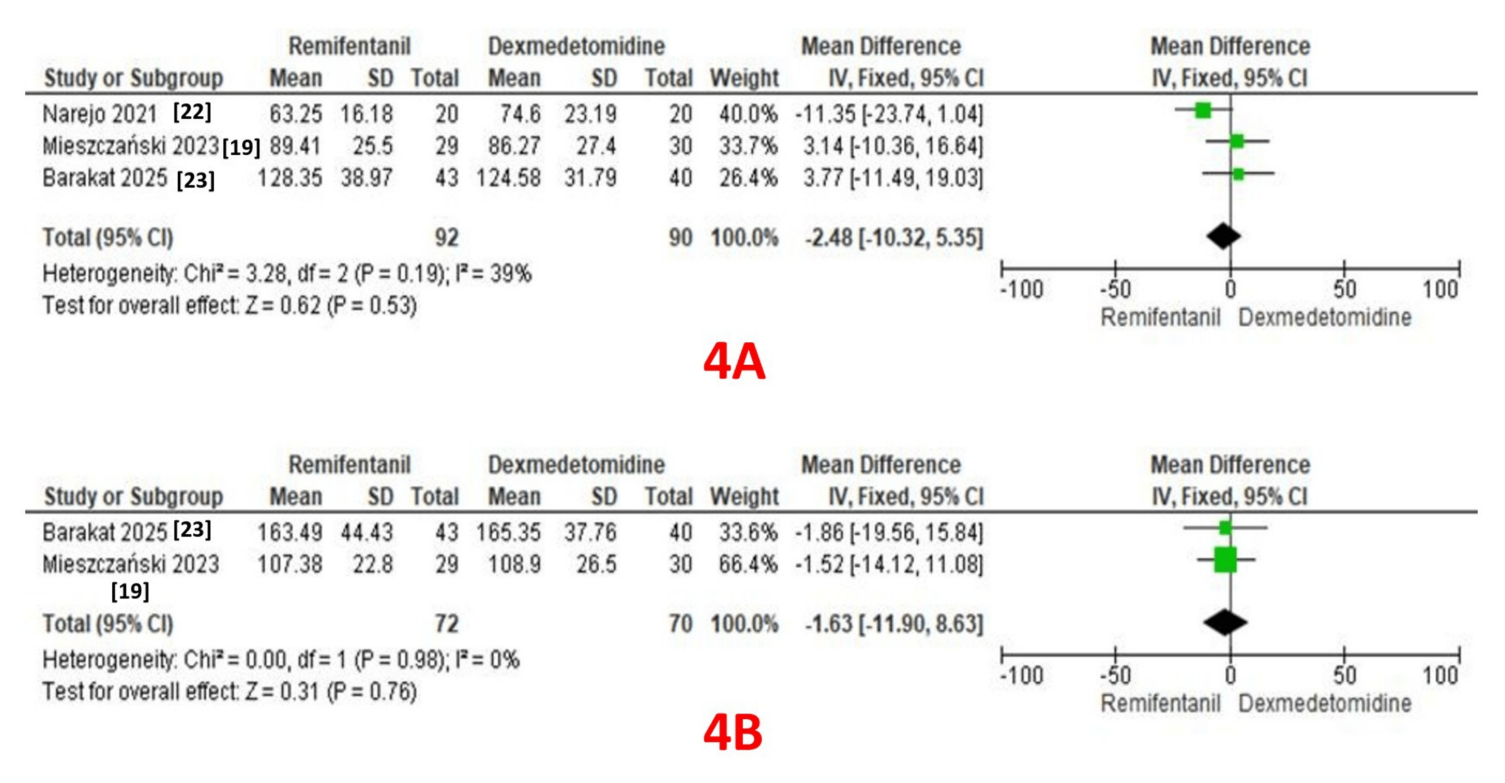
A: Forest plot comparing surgery duration between the two groups; B: Forest plot comparing anesthesia duration between the two groups.

Three studies reported surgery duration as an outcome (92 patients in the remifentanil group and 90 patients in the dexmedetomidine group) [19, 22, 23]. A pooled analysis revealed comparable durations in both groups (MD: -2.48; 95% CI: -10.32 to 5.35, P = 0.53). A fixed-effect model revealed moderate heterogeneity (I² = 39%) (Figure 4A).

Anesthesia Duration

Two studies reported anesthesia duration as an outcome (72 patients in the remifentanil group and 70 patients in the dexmedetomidine group) [19, 23]. A pooled analysis revealed comparable durations in both groups (MD: -1.63; 95% CI: -11.0 to 8.63, P = 0.76). A fixed-effect model revealed no heterogeneity (I² = 0%) (Figure 4B).

TSA Findings

**Figure 5:**
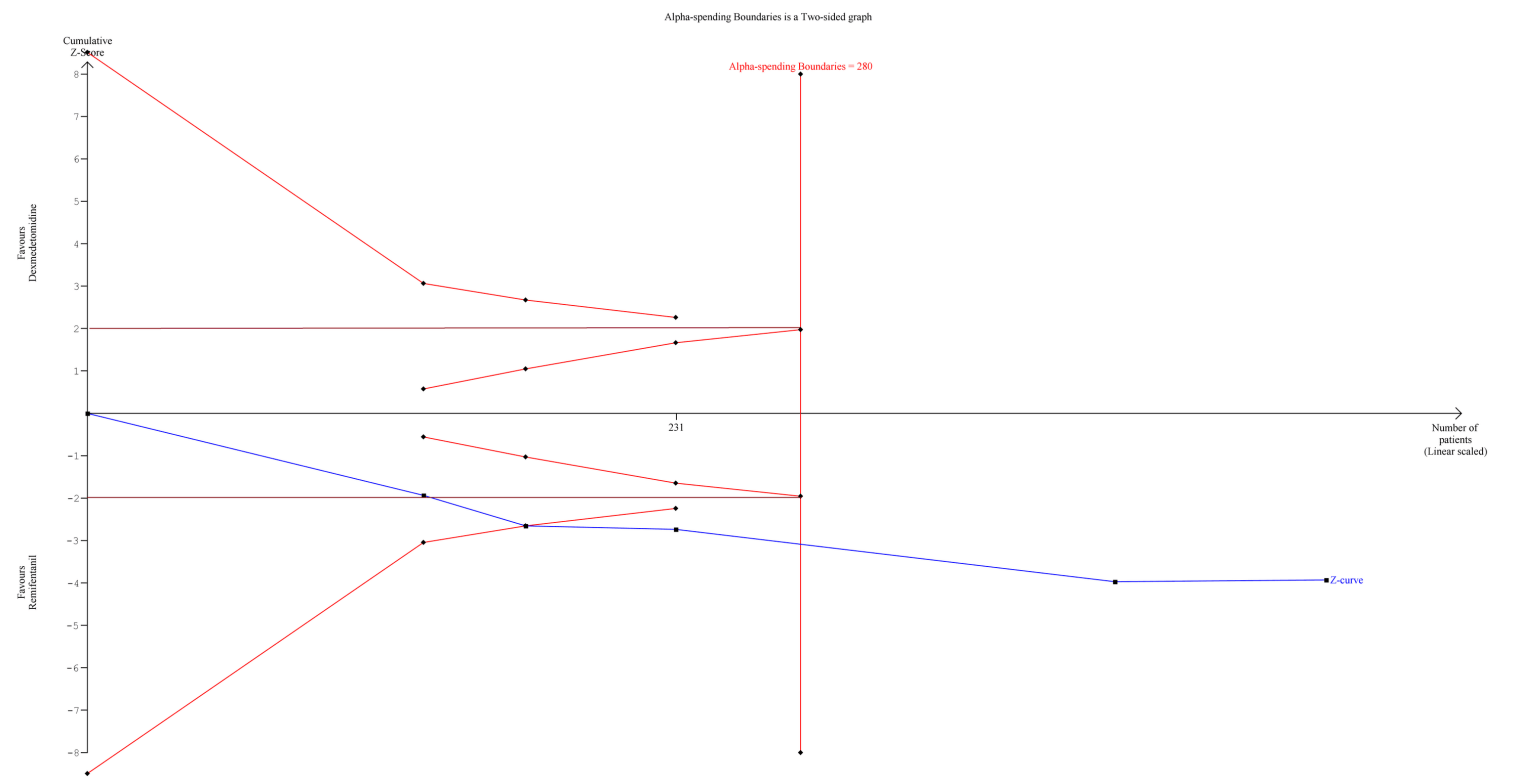
Trial sequential analysis comparing PONV between the two groups. PONV: Postoperative nausea/vomiting.

We performed TSA for only one outcome, PONV, as it demonstrated a significantly lower incidence in the dexmedetomidine group. TSA revealed that the information size (n = 231) reached 82.5% of the estimated RIS (n = 280), which was not substantially lower. As the cumulative Z-score curve crossed the conventional boundary or the trial sequential monitoring boundary, statistical significance was achieved (Figure 5).

Sensitivity Analysis

Sensitivity analysis was performed by removing one study at a time for each outcome. We found that there was no change in the statistical significance of the pooled results.

Publication Bias

Publication bias using a funnel plot was not assessed, as the number of articles included in the quantitative analysis was fewer than 10.

Discussion

Summary of Results

This systematic review and meta-analysis compared the analgesic efficacy and safety of remifentanil with dexmedetomidine as adjuncts to general anesthesia in adult patients undergoing bariatric and metabolic surgeries. Notably, this is the first review to compare an opioid with a non-opioid adjunct in bariatric surgical patients. The overall RoB in the included studies was low, while the GRADE quality of evidence ranged from moderate to low. A pooled analysis revealed that analgesic efficacy, assessed by 24-hour opioid consumption and pain scores in the recovery room, was comparable between the two groups. However, the incidence of PONV was significantly lower in the dexmedetomidine group, a finding supported by the TSA, despite a lower RIS. The overall heterogeneity among the included studies for various outcomes was low. Utilizing only RCTs for the review was a strength of our work.

The intraoperative infusion of remifentanil has been successfully used in various surgeries. Its short-acting nature and rapid recovery profile make it beneficial for certain procedures [24]. However, some studies have raised concerns about rapid tolerance, opioid-induced hyperalgesia, and an increased need for rescue analgesia in the form of opioids, leading to higher rates of postoperative adverse events such as PONV, drowsiness, and constipation. In a quantitative review of 85 studies involving 13,057 patients, Komatsu R et al. concluded that, compared to other short-acting opioids (fentanyl, alfentanil, or sufentanil), remifentanil facilitated faster recovery after general anesthesia [25]. However, the incidence of PONV was comparable, and the incidence of shivering was twice as high as with other opioids. They also mentioned that remifentanil does not confer any additional advantages over other opioids in prolonged or major surgeries.

In a systematic review by Al-Hassan A et al., postoperative pain and opioid use were compared in patients who received either remifentanil or dexmedetomidine [26]. The authors concluded that while remifentanil led to rapid onset of analgesia and faster recovery, it also resulted in increased postoperative opioid requirements. On the contrary, dexmedetomidine was associated with slower recovery and prolonged extubation but resulted in lower postoperative pain scores with significant opioid-sparing effects. In another systematic review, the authors evaluated the safety and efficacy of remifentanil compared to other agents, including sufentanil and dexmedetomidine [27]. Owing to significant heterogeneity between the interventional and control groups, the authors concluded that the current evidence was equivocal about the intraoperative use of remifentanil in bariatric surgeries. Opioid-sparing and opioid-free anesthesia (OFA) are gaining popularity in current clinical practice and are increasingly encouraged across specialties [28,29]. Dexmedetomidine has emerged as a safe and effective opioid-sparing medication, offering perioperative benefits such as reduced PONV and fewer adverse effects commonly associated with opioids. In a systematic review, Wang G et al. concluded that intraoperative dexmedetomidine use significantly reduced PONV and pruritus compared to placebo [30]. Several reviews have also confirmed that, when used as an adjunct, dexmedetomidine improves pain scores, decreases opioid consumption, lowers the incidence of PONV, and reduces postoperative delirium [31,32]. A single-center experience with a meta-analysis by Chang PC et al. further supported these findings, concluding that perioperative dexmedetomidine improves postoperative pain control and reduces overall PONV compared to its absence [33].

Subramaniam T et al. conducted a systematic review investigating the efficacy of dexmedetomidine in reducing PONV in patients undergoing bariatric surgery [34]. Based on 13 RCTs, the authors concluded that dexmedetomidine was more effective as PONV prophylaxis when the duration of surgery was less than 120 minutes. Our review findings align with this conclusion. In another systematic review and meta-analysis, Xu N et al. compared remifentanil and dexmedetomidine for controlled hypotension in certain surgeries. While both agents provided comparable hypotensive effects, dexmedetomidine was associated with better pain scores, less pruritus, and lower PONV compared to remifentanil [35]. However, the time to extubation was shorter in the remifentanil group. In a retrospective study of 134 patients undergoing bariatric surgery, Nam SW et al. compared recovery characteristics between those who received intraoperative remifentanil or dexmedetomidine [36]. They concluded that dexmedetomidine was associated with better pain scores, lower PONV, and reduced analgesic requirements in the immediate postoperative period. Singh PM et al. performed a meta-analysis with trial sequential analysis to investigate the perioperative analgesic profile of dexmedetomidine infusions in morbidly obese patients undergoing bariatric surgery [37]. Their analysis included six studies comparing dexmedetomidine with either an opioid (fentanyl or morphine) or placebo (saline). The results showed that patients in the dexmedetomidine group had lower opioid consumption during both the immediate and extended recovery phases, better pain control, and lower PONV. In a clinical study, Clanet M et al. compared a multimodal OFA technique to a multimodal opioid-based strategy during bariatric surgery [20]. They found no significant difference in postoperative morphine consumption between the groups. While the overall quality of recovery was similar, the OFA group experienced a lower incidence of PONV.

The strength of this review is that all included studies in the analysis were RCTs. However, some had small sample sizes, which could have influenced the findings. Despite this, most RCTs demonstrated an overall low RoB, further strengthening this review. Additionally, the TSA demonstrated statistical significance for PONV, confirming that dexmedetomidine was superior in reducing its incidence, although the RIS was 82.5%, which is not very low. Several important outcomes, such as length of stay (recovery room, hospital), patient satisfaction, and cost efficiency, were either inconsistently reported or not reported at all, preventing their analysis. Further studies should address these factors to provide clinicians with a more comprehensive comparison of medications.

## Conclusions

Based on the findings of this systematic review and meta-analysis, we conclude that remifentanil and dexmedetomidine offer comparable analgesic efficacy, as reflected by 24-hour opioid consumption and immediate postoperative pain scores, when used as adjuncts to general anesthesia in bariatric and metabolic surgeries. Dexmedetomidine was found to be associated with a significantly lower incidence of PONV compared to remifentanil. However, the current body of evidence is of moderate to low certainty. Further well-designed and adequately powered studies are warranted to determine the optimal anesthetic adjunct in this patient population.
